# Transcriptomic Response to *Perkinsus marinus* in Two *Crassostrea* Oysters Reveals Evolutionary Dynamics of Host-Parasite Interactions

**DOI:** 10.3389/fgene.2021.795706

**Published:** 2021-12-03

**Authors:** Jiulin Chan, Lu Wang, Li Li, Kang Mu, David Bushek, Yue Xu, Ximing Guo, Guofan Zhang, Linlin Zhang

**Affiliations:** ^1^ CAS and Shandong Province Key Laboratory of Experimental Marine Biology and Center of Deep Sea Research, Center for Ocean Mega-Science, Institute of Oceanology, Chinese Academy of Sciences, Qingdao, China; ^2^ Laboratory for Marine Biology and Biotechnology, Qingdao National Laboratory for Marine Science and Technology, Qingdao, China; ^3^ University of Chinese Academy of Sciences, College of Marine Science, Beijing, China; ^4^ Haskin Shellfish Research Laboratory, Department of Marine and Coastal Sciences, Rutgers University, Port Norris, NJ, United States

**Keywords:** oyster, comparative transcriptomics, dermo disease, innate immune response, host-parasite, interaction, gene expansion, adaptation

## Abstract

Infectious disease outbreaks are causing widespread declines of marine invertebrates including corals, sea stars, shrimps, and molluscs. Dermo is a lethal infectious disease of the eastern oyster *Crassostrea virginica* caused by the protist *Perkinsus marinus*. The Pacific oyster *Crassostrea gigas* is resistant to Dermo due to differences in the host-parasite interaction that is not well understood. We compared transcriptomic responses to *P. marinus* challenge in the two oysters at early and late infection stages. Dynamic and orchestrated regulation of large sets of innate immune response genes were observed in both species with remarkably similar patterns for most orthologs, although responses in *C. virginica* were stronger, suggesting strong or over-reacting immune response could be a cause of host mortality. Between the two species, several key immune response gene families differed in their expansion, sequence variation and/or transcriptional response to *P. marinus*, reflecting evolutionary divergence in host-parasite interaction. Of note, significant upregulation of *inhibitors of apoptosis* (*IAPs*) was observed in resistant *C. gigas* but not in susceptible *C. virginica*, suggesting upregulation of *IAPs* is an active defense mechanism, not a passive response orchestrated by *P. marinus*. Compared with *C. gigas*, *C. virginica* exhibited greater expansion of *toll-like receptors* (*TLRs*) and positive selection in *P. marinus* responsive *TLRs*. The C1q domain containing proteins (*C1qDCs*) with the galactose-binding lectin domain that is involved in *P. marinus* recognition, were only present and significantly upregulated in *C. virginica*. These results point to previously undescribed differences in host defense genes between the two oyster species that may account for the difference in susceptibility, providing an expanded portrait of the evolutionary dynamics of host-parasite interaction in lophotrochozoans that lack adaptive immunity. Our findings suggest that *C. virginica* and *P. marinus* have a history of coevolution and the recent outbreaks may be due to increased virulence of the parasite.

## Introduction

Marine diseases can be caused by a variety of factors, such as parasite infections, biological toxins and environmental stress (Bossart, 2007; Burge et al., 2014; Wilson and Ho, 2015). Environmental stress caused by climate change and other human activities contributes to the development of marine diseases. Global warming has caused range extension of some parasites (Ford, 1996; Studer et al., 2010), and ocean acidification may weaken the immune defense of calcifying hosts (Bibby et al., 2008). Human activities have facilitated rapid transmission of some marine infectious diseases (Harvell, 1999; Harvell et al., 2004; Guo and Ford, 2016). Outbreaks of marine infectious disease have become more frequent and severe in recent decades (Groner et al., 2016; Sutherland et al., 2016).

Infectious diseases can cause mass mortalities of diverse marine organisms, affecting their abundance, changing community structure, and threatening the health of marine ecosystems (Harvell et al., 2004; Tim, 2007). They may impact major fishery and aquaculture species and cause immeasurable economic losses. Molluscs are a major group of marine animals, many of which are economically important as major fishery and aquaculture species (Guo et al., 1999; Shumway, 2021). Molluscan populations, fisheries and aquaculture are seriously affected by infectious disease outbreaks that have become more frequent and severe due to climate changes and human activities (Guo and Ford, 2016).

Dermo is a lethal disease of the eastern oyster *Crassostrea virginica* caused by the protozoan parasite *Perkinsus marinus*. The intracellular parasite is transmitted directly from oyster to oyster through the water. The infection is characterized by hemocyte infiltration followed by tissue fibrinolysis and blockages of hemolymph vessels, leading to inhibition of gonadal development and death of the oyster by emaciation (Faisal et al., 1999). Dermo causes extensive mortalities of the eastern oyster along the Atlantic coast of United States and threatens both aquacultural and wild populations (Levine, 1978; Reece et al., 2008). Mortality events associated with this disease cause tremendous losses in the oyster industry, and often wipe out up to 100% of harvestable naive stocks. Such mass mortalities negatively impact water quality and the overall health of the ecosystem (Newell, 1988). While *P. marinus* is a highly successful parasite of *C. virginica*, the Pacific oyster *Crassostrea gigas*, a sister species from Asia, is resistant to *P. marinus* infections (Meyers et al., 1991). The parasite can infect *C. gigas* at low intensity but does not cause serious pathologies or mortalities. How sister species without adaptive immunity differ in their susceptibility to the same pathogen is of fundamental interest. The difference in Dermo resistance between two *Crassostrea* oysters provides a good opportunity to study the evolution of host-parasite interactions in organisms without adaptive immunity.

The complex interaction between the host and parasite is fundamental to the infection process, the outcome of which often determines life or death of the host or parasite. The evolutionary race between a host and its parasites as best characterized by the Red Queen hypothesis is intense and everlasting (Van Valen, 1973). The ability of a particular pathogen to cause disease is determined by its virulence, and the total “cards of virulence” possessed by a pathogen determine its relative capacity to induce disease within a host (Winnicki, 2010; Kim et al., 2016). Host organisms need to evolve sophisticated defense mechanisms against parasites that constantly invent new ways to infect. Host-pathogen interactions are often dynamic, and the disease outcome is commonly dependent on multiple factors including the state of the host, the pathogenic potential of the pathogen and environmental conditions or stressors (Allam et al., 2013; Debasish et al., 2021).

While bivalve molluscs do not have the canonical adaptive immune system, they have evolved effective host-defense mechanisms to cope with biotic and abiotic stress (Guo et al., 2015; Wang et al., 2018). The bivalve host-defense system includes two major components, humoral and cellular immune responses. On the molecular level, the humoral immunity starts from recognizing conserved microbial-associated molecular patterns (MAMPS) and pathogen-associated molecular patterns (PAMP) (Aladaileh et al., 2007). Upon recognition, the pathogen-associated pattern recognition receptors (PRRs) activate immune signaling pathways and antimicrobial effectors (Akira et al., 2006). The cellular immune responses begin from phagocytosis and encapsulation by hemocytes, and then trigger the release of enzymes and oxygen metabolites (Wang et al., 2018). Oxidative bursts not only have a cytotoxic effect on invading pathogens but also serve as signals that activate further defense reactions (Anderson, 1999; Goedken et al., 2005). Besides immune reactions, apoptosis (Zhang et al., 2011; Qu et al., 2015; Li et al., 2017) and authophage (Moreau et al., 2015) also play an important role in host defense of bivalves.

Despite the devastating impact of Dermo, the molecular mechanisms of the host-parasite interplay between oysters and *P. marinus* are still largely unknown (Anderson, 1996). Tanguy et al. (2004) identified candidate genes from several pathways that were upregulated in response to *P. marinus* infection in *C. virginica* and *C. gigas*, including *toll-like receptors*, *galectin*, and genes involved in energy metabolism and metal binding. A galectin of *C. virginica* has been shown to function as a hemocyte receptor that recognizes *P. marinus* and facilitates phagocytosis (Tasumi and Vasta, 2007). Using a 12 K oligonucleotide microarray, Wang et al. (2010) revealed that genes involved in antimicrobial defense, pathogen recognition and uptake, oxidative stress and apoptosis are differentially expressed under *P. marinus* infection. Transcriptomic analyses in *C. virginica* identified a large set of differentially expressed genes and the upregulation of genes for proteolysis regulation and oxidation-reduction processes in resistant families (Proestou and Sullivan, 2020). Models have been developed to simulate and predict *P. marinus* transmission in *C. virginica* populations (Powell et al., 2011; Bidegain et al., 2017). It has been suggested that *C. gigas* may be more effective in degrading *P. marinus* than *C. virginica* (La Peyre et al., 1995), although the molecular mechanisms that underpin its effective defense are not understood.

In this study, we compared transcriptomic response to *P. marinus* infection in the susceptible eastern oyster with that of the resistant Pacific oyster, to better understand the evolutionary dynamics of host-parasite interactions. Comparative analyses revealed specific dynamic and orchestrated expression of a large set of innate immune genes in both species. Comparing infected gene expression profiles against *P. marinus* infection between two oysters identified previously undescribed differences in host defense, which may underpin differences in susceptibility and point to new mechanisms for the evolution of disease resistance. These results contribute to our understanding of host-defense and adaptation, as well as the development of disease-resistant stocks for aquaculture.

## Materials and Methods

### Experimental Oysters, *P. marinus* Challenge and Sample Collection

**FIGURE 1:**
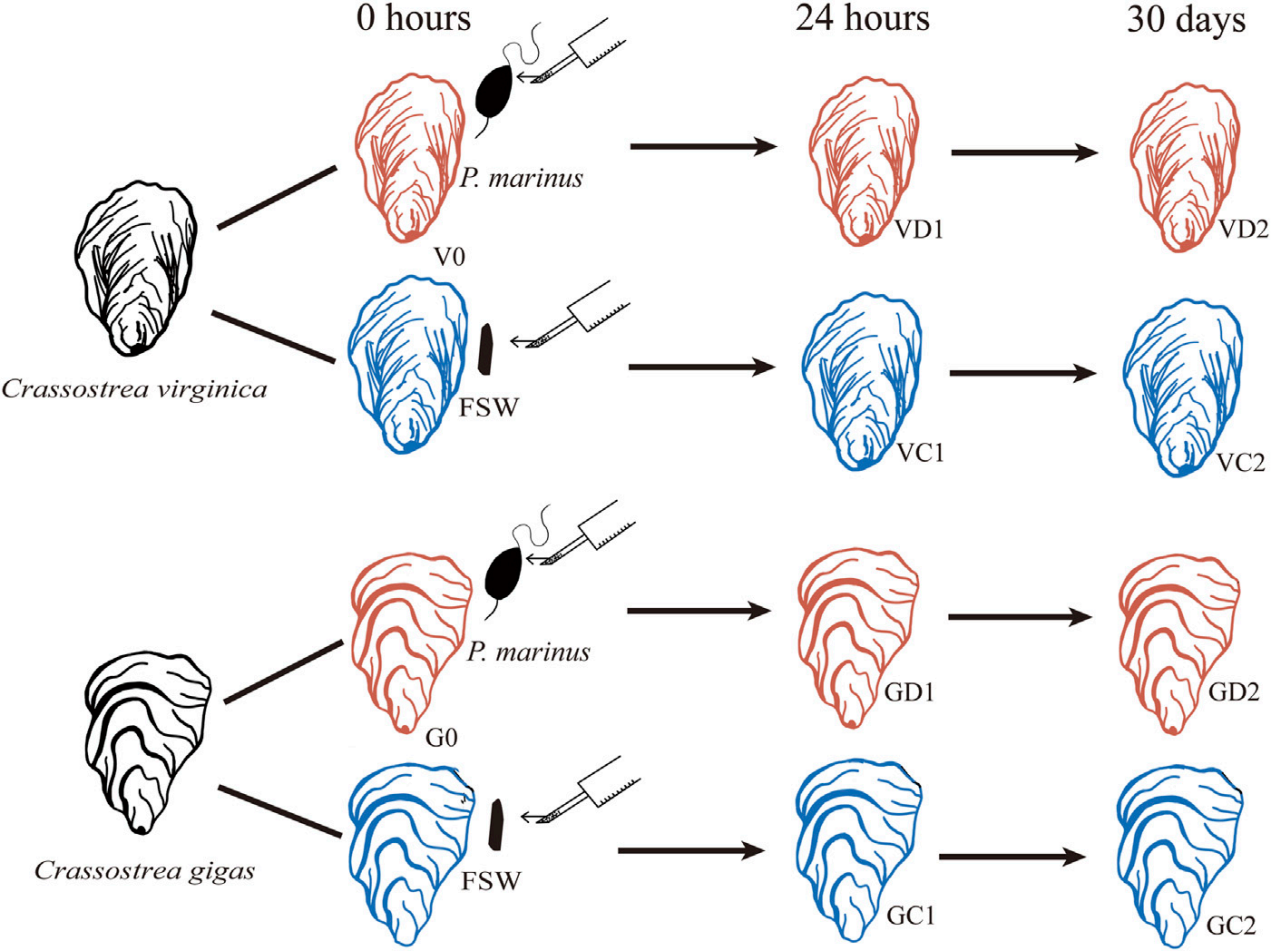
Schematic diagram of experimental design. FSW, filtered seawater.

The eastern and Pacific oysters used in this study were obtained from oyster farms in Maine and Washington, respectively. Oysters were brought to the lab and acclimated for 24 h in 1 μm filtered seawater at 23°C before the challenge experiment. All oysters were notched at the ventral edge of the shell adjacent to the gills in preparation for shell cavity inoculation after previous studies using *P. marinus* harvested from infected oysters (Chintala et al., 2002; Ford et al., 2002). For the challenged group, the shell cavity of each oyster was inoculated with 1.2 × 10^6^ *P. marinus* cells in 100 μL of seawater fortified with 100 U penicillin and 100 μg of streptomycin mL^−1^ (pen/strep). The control oysters were injected with 100 μL filtered seawater (FSW) similarly fortified with pen/strep (Figure 1). At the time of injection, ten oysters from each species were sampled, and a piece of gill was preserved in RNAlater as Time 0 samples. After injection, a wide rubber band was placed around each oyster to cover the notch and to minimize ejection of the inoculum. Eight hours after the injection, the oysters were returned to seawater and fed every day with algal paste. Seawater was changed twice per week. The infection by *P. marinus* was determined with Ray’s Fluid Thioglycollate Medium (RFTM, Bushek et al., 1994). At 24 h and 30 days post injection, ten oysters were sampled from challenged and control groups of each species by fixing a piece of gill in RNAlater. Samples were stored at −80°C before RNA extraction.

### RNA Isolation and Library Construction

Total RNA of each sample was extracted using the Trizol-chloroform extraction method. The RNA quality and concentration were checked by Qubit™ RNA BR Assay Kit (Thermo Fisher Scientific) and Agilent Bioanalyzer 2100. All extracted samples had an A260/280 ratio greater than 1.8. RNA from the ten oysters was extracted separately and then pooled with equal amounts into one sample for library construction. RNA-sequencing libraries were constructed using the NEBNext Ultra RNA Library Prep Kit, following the manufacturer’s instructions. Briefly, 1 mg of total RNA from each sample was used for the double stranded cDNA synthesis. Then, size selection was performed using a 2% agarose gel to remove self-ligated adaptors, generating cDNA libraries with an average size of 200–250 bp. RNA sequencing of prepared libraries was carried out on high-throughput Illumina sequencing platforms. Libraries were quantified, multiplexed, and sequenced as 49 bp single-end reads. A total of 159 M reads were generated in this study (Supplemenatary Table S1).

### Data Analysis, Identification and GO Enrichment Analysis of Differentially Expressed Genes

FastxToolkit pipeline (http://hannonlab.cshl.edu/fastx_toolkit/index.html) was used to process the raw reads to evaluate sequencing quality, remove low-quality reads (length threshold <50 bp and quality threshold <20), adaptor sequences, poly-N and known non-coding RNAs. The obtained clean reads were then mapped to the genomes of *C. virginica* (v3.0; NCBI Bioproject PRJNA379157) and *C. gigas* (v9; NCBI Bioproject PRJNA70283) by Tophat2 software (v2.1.1, Trapnell et al., 2009). Gene expression levels were measured by fragments per kilobase of exon per million fragments mapped (FPKM) using HT-seq (Anders et al., 2015). The differentially expressed genes (DEGs) were identified with the edgeR tool of R programming language with the threshold value |log_2_FC| ≥ 1 (multiple of Fold change, FC: difference) and FDR ≤ 0.05. The gene ontology (GO) enrichment analysis of DEGs was conducted with Blast2GO software. The annotated results with corrected FDR ≤ 0.05 were selected as the enrichment functions. Fisher’s LSD was used to test significant enrichment when the number of genes in a GO term was less than 5, and χ_2_ test was used when the number of genes ≥5.

### Identification of Orthologous Genes Between *C. virginica* and *C. gigas*

OrthoFinder (Emms and Kelly, 2015) was used to identify orthologous genes between *C. virginica* and *C. gigas* with the following steps: 1) all-vs-all search through BLAST for potential orthologous genes with evalue ≤ 1e-3 as the threshold; 2) standardize the score of BLAST bit based on gene length and phylogenetic analysis; 3) use Reciprocal Best length-Normalised Hit (RBNHs) to determine the threshold of sequence similarity of homologous groups; and 4) Markov clustering (MCL) gene clustering and orthologous grouping.

### Identification of Immune-Related Genes

We used domain prediction and sequence homology search to annotate genes that are potentially involved in immune response in *C. virginica* and *C. gigas*. First, InterProScan (Quevillon et al., 2005) and Pfam (Mistry et al., 2021) were used to annotate genes and gene models with characteristic immune domains or motifs. Second, BLASTP package (Mahram and Herbordt, 2015) was used to search against oyster gene datasets with canonical immune genes from model organism as inquiries. Third, TBLASTN package (Gertz et al., 2006) and Genewise (http://www.ebi.ac.uk/Tools/Wise2/index.html) were used to predict genes from genome sequences. Immune gene models predicted from these three methods were integrated to the primary immune geneset. Then, all the candidate gene models annotated with immune function by blast against NCBI nr database and predicted with immune domains by SMART (http://smart.embl-heidelberg.de/) were manually checked, resulting in the final immune geneset.

### Phylogenetic Analysis

Multiple sequence alignments were performed using ClustalW with default parameters and the resulting alignments were refined with trimAl (Capella-Gutierrez et al., 2009). Phylogenetic trees were constructed with maximum likelihood (ML) analytical approaches. MEGA and PHYML were used to construct the NJ and ML trees, respectively. The robustness of the inferred trees was assessed using bootstrapping 1,000 in the phylogenetic tree.

PAML (v4) was used for comparing the rate per site of dN (non-synonymous) to the rate per site of dS (synonymous) mutations. The recommended subset of four M-series models of M1a (nearly neutral), M2a (positive selection), M7 (beta) and M8 (beta & ω) coupled with Bayesian Empirical Bayes (BEB) methods (Yang et al., 2005) were implemented. The Log-likelihood values (lnL) of M2a-M1a and M8-M7 were from explicit tests for the presence of positively selected sites. The *p* values were corrected by a multiple testing correction method (Benjamini and Hochberg, 1995). Furthermore, the probabilities of sites under positive selection were assessed by their posterior probabilities calculated with the BEB method. The amino acid site would be considered as a positively selected site if the value of dN/dS > 1 appears in the LRT and posterior probability exceeds 90% (Lan et al., 2018). Finally, SWISS-MODEL (http://swissmodel.expasy.org/) was used to locate and visualize the positively selected sites.

## Results

### *P. marinus* Challenge Experiment in the Oysters

No *P. marinus* was detected in *C. virginica* and *C. gigas* at Time 0, and in unchallenged control of either species on Day 30 (*n* = 10 for all). In the challenged groups, *P. marinus* was detected in 90% of *C. virginica* (*n* = 40) at an average infection intensity of 3.2 (∼160,938 *P. marinus* cells g^−1^ wet tissue, Choi et al., 1989), which is heavy and indicates that the challenge is successful. Infection was detected in all *C. gigas* (*n* = 15) but at a lower average infection intensity of 1.4 (∼11,202 *P. marinus* cells g^−1^ wet tissue, Choi et al., 1989), confirming a successful challenge and that *C. gigas* is resistant or less susceptible.

### Transcriptome Analyses of *C. virginica* Response to *P. marinus* Challenge

**FIGURE 2:**
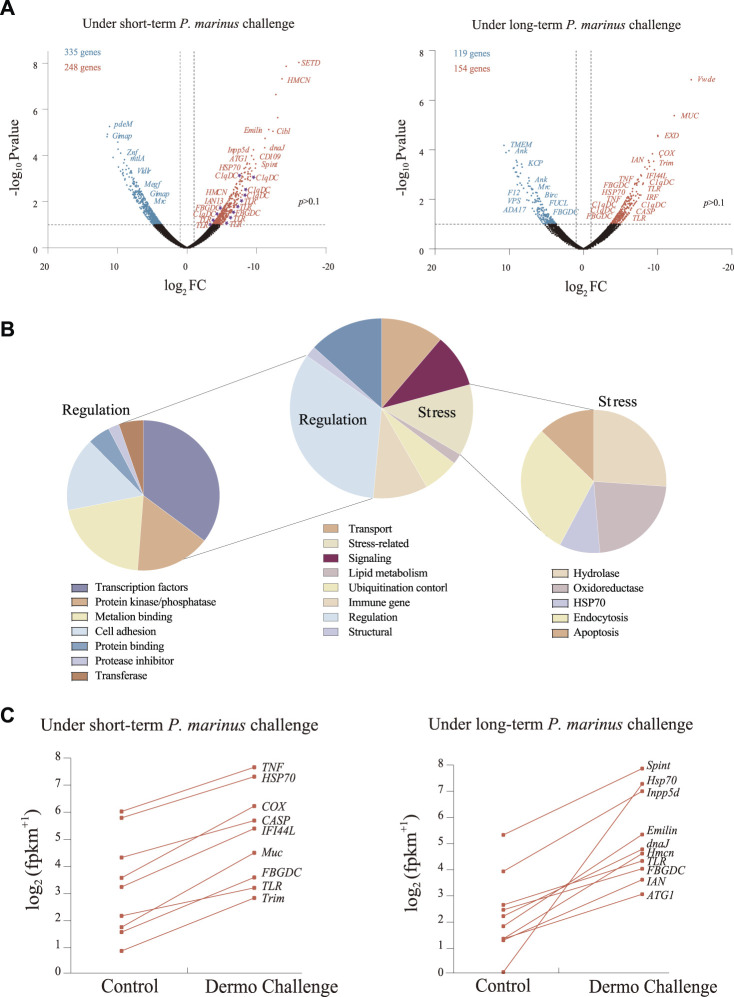
Transcriptional changes induced by *P. marinus* in *C. virginica.* **(A)**, Volcano plots of transcriptional change under short-term and long-term *P. marinus* challenge. Blue points represent downregulated genes, red points represent upregulated genes, and black points represent non-differentially expressed genes. **(B)**, Functional annotation of differentially expressed genes under *P. marinus* challenge. **(C)**, Immune-related genes significantly up-regulated after dermo challenge.

After filtering and cleaning, 65,537,432 high-quality reads were mapped to the reference genome for *C. virginica*, averaging 10 to 14 million per sample (Supplementary Table S1). The mapping ratio ranged from 80.8 to 84.2%. Of the 39,505 protein-coding genes in the *C. virginica* genome, 28,918 genes showed expression levels of FPKM ≥ 1. Overall, 759 genes were differentially expressed in oysters challenged with *P. marinus* (VD1 and VD2) compared with unchallenged controls (V0, VC1, and VC2) (Supplemenatary Table S2), including 583 DEGs from short-term challenge (24 h) and 273 DEGs from long-term challenge (30 days) (Figure 2A). Of these, 248 and 154 DEGs were up-regulated while 335 and 119 genes were down-regulated under short- and long-term *P. marinus* challenge, respectively (Figure 2A).

Functional analysis showed that the 759 DEGs induced by *P. marinus* challenge were mostly involved in transport, stress, signaling, lipid metabolism, ubiquitination control, immune response, regulation and structure (Figure 2B). The “regulation” term was further classified into categories of transcription factors, protein kinase/phosphatase, metal ion binding, cell adhesion, protein inhibitor and transferase. Similarly, the “stress” term included sub-categories of hydrolase, oxidoreductase, HSP70, endocytosis and apoptosis. The up-regulation of genes related to “regulation” and “stress” terms indicated that genes related to stress response system and metabolism were largely recruited under challenge.

A large number of genes encoding immune receptors (e.g., *TLRs*, *C1qDCs*, *FBGDCs*) and effectors were significantly upregulated during pathogen infection (Figures 2A,C), revealing their important role in the response to *P. marinus* challenge in *C. virginica*.

### Comparative Transcriptomic Analyses of Resistant *C. virginica* and Susceptible *C. gigas*

**FIGURE 3:**
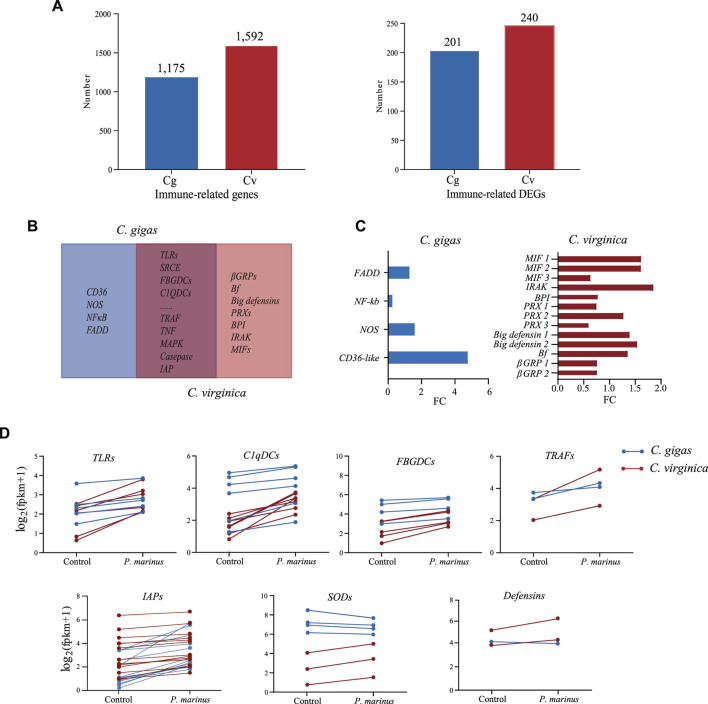
Comparative transcriptome analysis between *C. gigas* and *C. virginica* under *P. marinus* challenge. **(A)**, Number of immune-related genes identified **(Left)** and number of immune-related genes differentially expressed **(Right)** in *C. gigas* (Cg) *and C. virginica* (Cv). **(B)**, Venn diagrams of differentially expressed immune genes under *P. marinus* challenge between the two oyster species. **(C)**, Unique and differentially expressed immune genes induced by *P. marinus* in two oyster species. **(D)**, Transcriptional change of differentially expressed immune genes shared by two oyster species, including pattern recognition receptors (e.g., *TLRs*) and immune effectors (e.g., *TRAFs*).

To understand the differences in host defense between the two species, we first annotated immune related genes in the two genomes: 1,175 in *C. gigas* and 1,592 in *C. virginica* (Figure 3A). Of these, 201 in *C. gigas* and 240 in *C. virginica* were differentially expressed during *P. marinus* infection (Figure 3A). Most of these genes were shared by the two oyster species (Figure 3B). These shared DEGs mainly consisted of genes for immune recognition receptors, effectors, lectins, and other proteins related to host-parasite interaction that play important roles in preventing infection or improving defense against pathogens. In addition, genes coding for CD36, NOS, NF-κB, and FADD were differentially expressed in *C. gigas* but not in *C. virginica*. Meanwhile genes coding for βGRP, Bf, Big defensin, PRX, BPI, IRAK, and MIF were differentially expressed only in *C. virginica* (Figure 3C). The gene *Cvgal*, a galectin consisted of four carbohydrate recognition domains (CRDs) and a hemocyte receptor of *P. marinus* (Tasumi and Vasta, 2007), was only significantly upregulated by *P. marinus* infection in *C. virginica* (Supplementary Figure S1).

Although there was no significant difference in the numbers of differentially expressed immune-related genes between the two oyster species after *P. marinus* infection, the magnitude of up-/downregulation of the shared immune-related DEGs were different between the two oyster species. For instance, the upregulation of PRRs such as *TLRs*, *FBGDCs* and *C1qDCs* were much higher in *C. virginica* than that in *C. gigas* (Figure 3D). Similarly, immune adaptors (e.g., *TRAF*) and effectors (e.g., *SOD* and *Big defensin*) also showed stronger upregulation *in C. virginica* (Figure 3D). In contrast, stronger upregulation of *inhibitors of apoptosis* (*IAPs*) was observed in *C. gigas* than in *C. virginica* under *P. marinus* infection (Figure 3D). Thirteen of the expressed 23 *IAPs* showed 2-fold or more upregulation in *C. gigas* compared with only one of the 30 in *C. virginica*.

**FIGURE 4:**
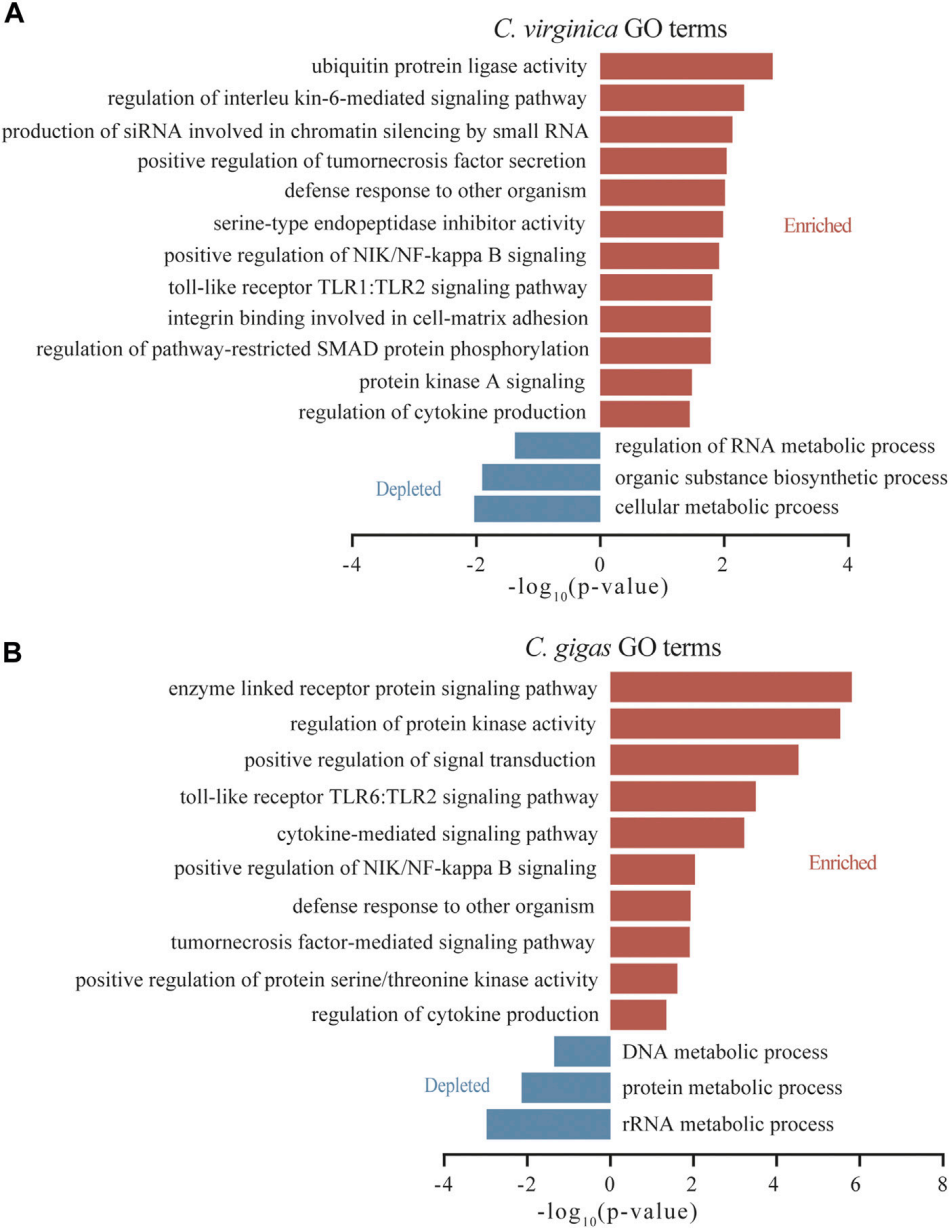
GO enrichment analysis of the differentially expressed genes after Dermo challenge between *C. virginica* **(A)** and *C. gigas* **(B)**. Red and blue columns represent functional enrichments of up-regulated and down-regulated genes, respectively.

Among DEGs induced by *P. marinus*, GO terms for “interleukin-6-mediated signaling pathway,” “production of siRNA involved in chromatin silencing by small RNA,” and “serine-type endopeptidase inhibitor activity” were significantly enriched in *C. virginica* (Figure 4A), while GO terms for “positive regulation of protein serine/threonine kinase activity” were significantly enriched in *C. gigas* (Figure 4B). Interestingly, TLRs were significantly enriched in both species, but for different TLR pathways: “TLR1:TLR2” in *C. virginica* versus “TLR6:TLR2” in *C. gigas* (Figure 4). This finding indicates that different types of *TLRs* may play different roles in responding to *P. marinus* in the two oyster species.

### Positive Selection in *P. marinus* Responsive TLRs in *C. virginica*

**FIGURE 5:**
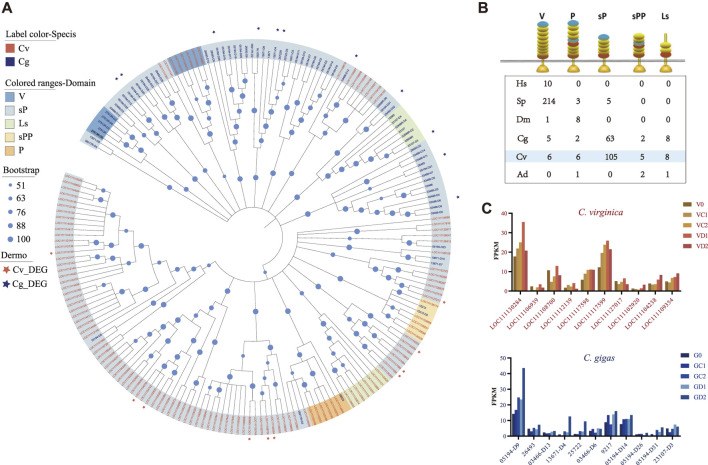
Expansion of TLRs and divergent expression patterns under *P. marinus* challenge between *C. gigas* and *C. virginica.* **(A)**, Phylogenetic tree constructed with the maximum likelihood method showing lineage-specific expansion of TLRs in *C. gigas* (red font) and *C. virginica* (blue font). Red and blue asterisks represent the differentially expressed TLRs in *C. gigas* and *C. virginica*, respectively. **(B)**, Comparison of TLRs with different domain architectures in: human (*Homo sapiens*-Hs), sea urchin (*Strongylocentrotus purpuratus*-SP), fruit fly (*Drosophila melanogaster*-Dm), Pacific oyster (*Crassostrea gigas*-Cg), Eastern oyster (*Crassostrea virginica-*Cv), and staghorn coral (*Acropora digitifera*-Ad). LRRCT in red, LRRNT in blue and LRR in yellow. Toll/interleukin-1 receptor (TIR) domains are shown as gold triangles. **(C)**, Diverse expression pattern of the differentially expressed TLRs, marked with asterisks in **(A)**, during *P. marinus* challenge in *C. gigas* **(Top)** and *C. virginica* **(Bottom).**

Given that the pattern recognition receptor genes (*TLRs*, *C1qDCs*, and *FBGDCs*) showed significant differential expression in the two oyster species under *P. marinus* challenge, we examined molecular evolutionary history of these gene families in the two species. The *TLRs* can be separated into five types based on domain structures: V-, P-, sP-, sPP-, and Ls-type. The sP-type was greatly expanded in oysters, and to a greater extent in *C. virginica* (105) than in *C. gigas* (63) (Figures 5A,B). All *P. marinus* responsive *TLRs* of *C. virginica* were different from those of *C. gigas* (Figure 5A). These results indicated that these *P. marinus* responsive *TLRs* were from different lineage-specific expansions in the two oyster species and may have different functions in their response to *P. marinus*. All differentially expressed *TLRs*, 10 in *C. virginica* and 11 in *C. gigas*, belonged to sP-type (Figures 5A,C).

**FIGURE 6:**
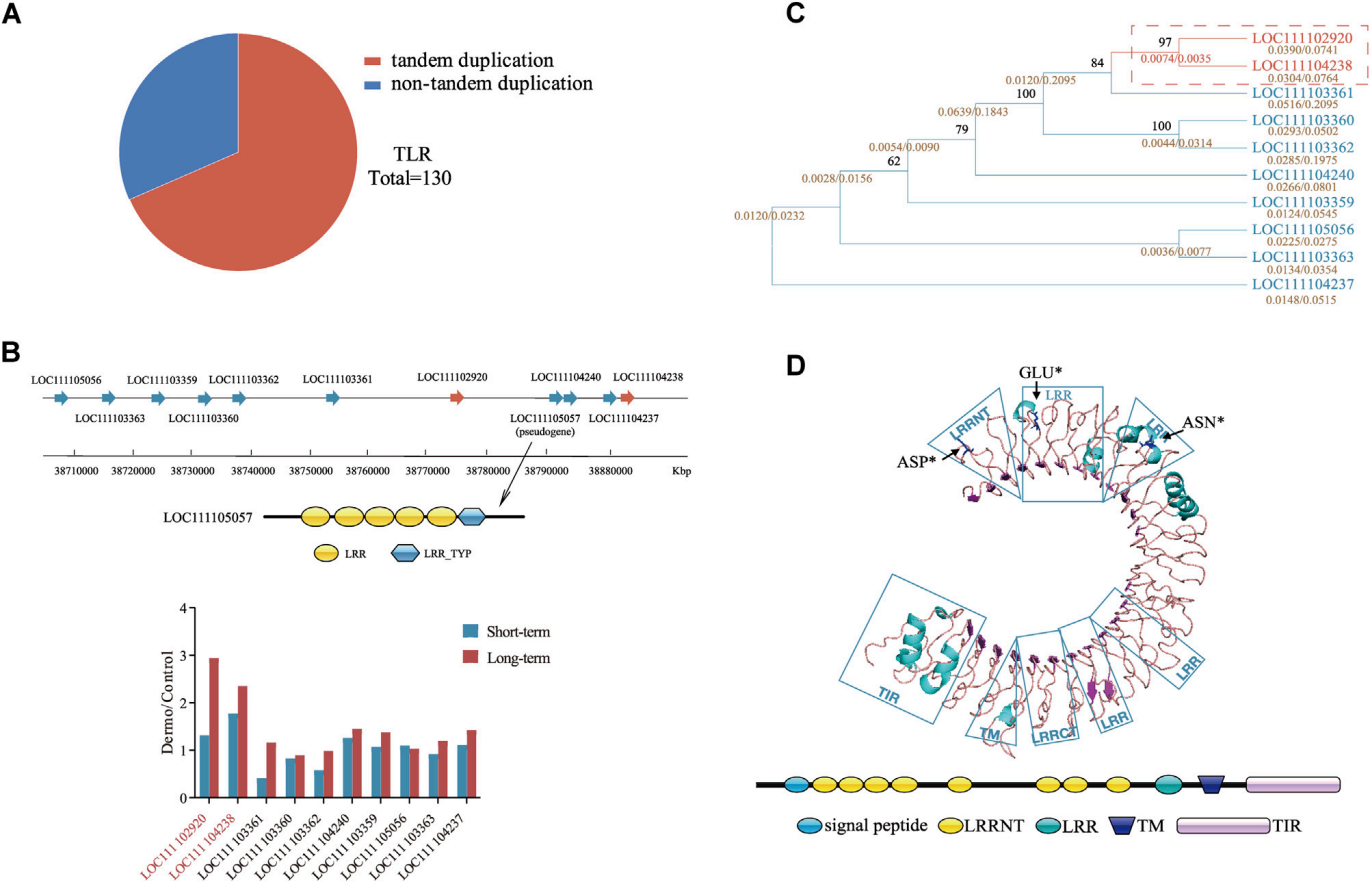
Tandem duplication and positive selection of TLRs in *C. virginica*. **(A)**, Tandem repeats as the major source of TLRs duplication in *C. virginica.* **(B)**, The largest tandem repeat cluster **(Top)** and divergent expression patterns **(Bottom)** of tandemly linked TLRs in NC_035786.1 under *P. marinus* challenge. The wide arrows represent TLR genes, and genes with red background are differentially expressed under *P. marinus* challenge. A pseudogene LOC111105057 was detected in this repeat cluster. **(C)**, Evolutionary relationships of duplicated TLRs. The calculated d_N_/d_S_ (ω) values (gold) and bootstrap values are shown for each branch. The branches with ω values >1.0 are marked with red rectangle. The differential genes under *P. marinus* challenge that are positively selected are boxed with red dash lines. **(D)**, Structural modeling of TLR depicting sites (blue) under positive selection. Positive selection sites (Asp^27^, Glu^62^, Asn^199^) are represented by black arrows.

Mapping of *TLRs* to reference genomes revealed that most of the *TLRs* expansion in *C. virginica* and *C. gigas* was due to tandem duplication. For instance, of the 130 *TLRs* of *C. virginica*, 89 (68.46%) were found in tandemly duplicated clusters (Figure 6A). The largest cluster in *C. virginica* was located on chromosome 7 (NC_035786.1) and consisted of 11 *TLRs* (Figure 6B). In addition, a pseudogene LOC111105057 was detected within this cluster, suggesting that these expanded *TLRs* experienced rapid divergence including functional loss. Furthermore, two *TLRs* (LOC111102920 and LOC111104238) in this cluster that were significantly upregulated after the *P. marinus* challenge (Figure 6B) were clustered together on the phylogenetic tree (Figure 6C). Examination of synonymous (*d*_*S*_) and nonsynonymous (*d*_*N*_) nucleotide substitutions among *TLRs* of the cluster detected positive selection signals in these two upregulated *TLRs* (Figure 6C). The positive selection sites Asp 27 (*p* < 0.05), Glu 62 (*p* < 0.05) and Asn 199 (*p* < 0.05) were located in the LRR and LRRNT regions (Figure 6D) and likely involved in ligand/pathogen recognition. Similar analysis was performed in *P. marinus* responsive *TLRs* of *C. gigas*, but no positive selection sites were detected (Supplementary Figure S2). These results suggested that positive selection observed in *P. marinus* responsive *TLRs* may be important for the evolution of host-parasite interaction between *C. virginica* and *P. marinus*.

### Upregulation of *C1qDC* With Galactose-Binding Lectin Domain by *P. marinus*

**FIGURE 7:**
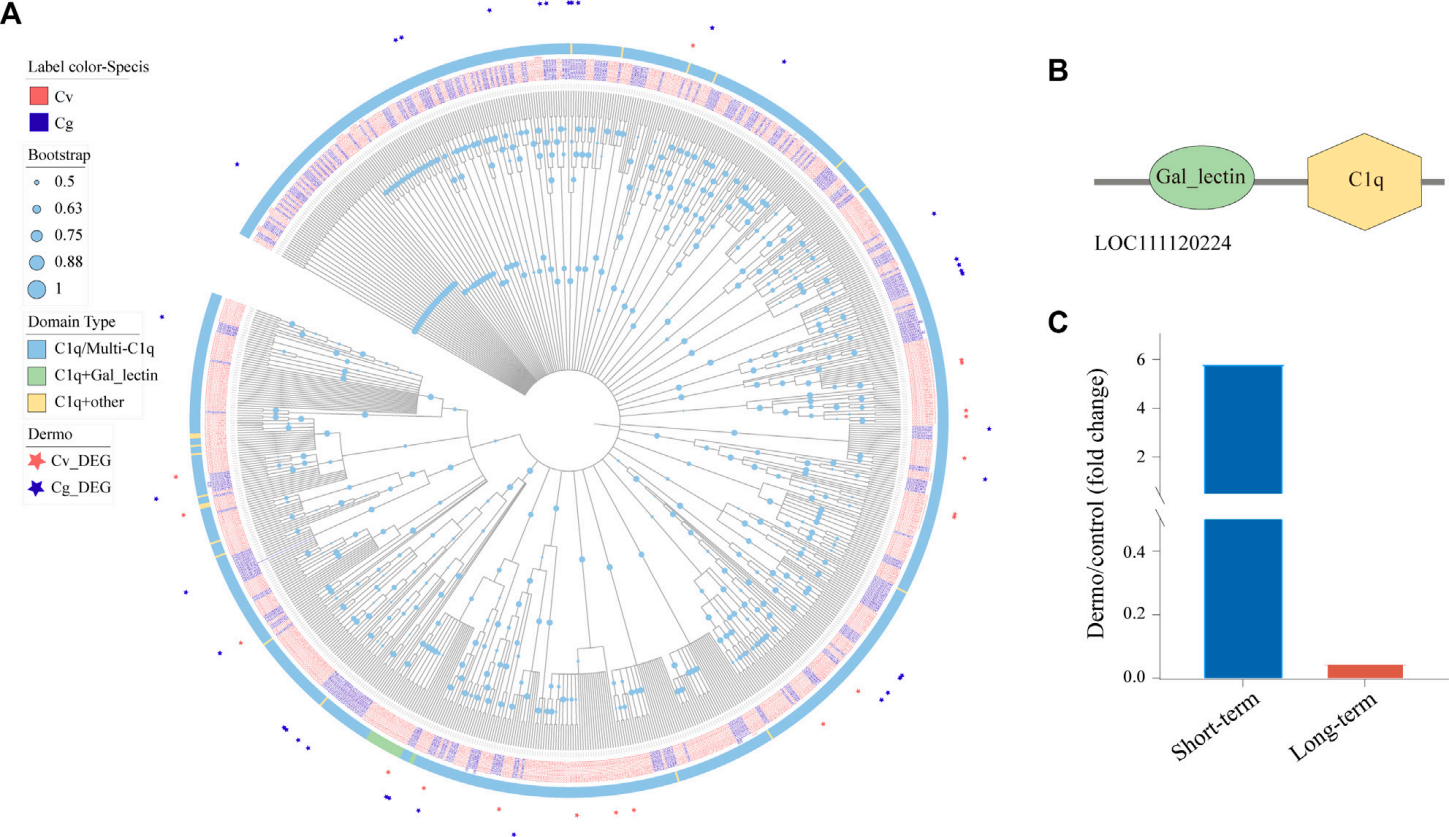
Expansion of C1qDCs and divergent expression patterns under *P. marinus* challenge between *C. virginica* and *C. gigas*. **(A)**, Phylogenetic tree constructed with the maximum likelihood method showing lineage-specific expansion of C1qDCs in *C. virginica* (red font) and *C. gigas* (gray font). Different color backgrounds represent different types of C1qDC domain composition, and the red and gray asterisks represent differentially expressed C1qDCs under *P. marinus* challenge in *C. virginica* and *C. gigas*, respectively. **(B)**, Domain composition of the “galactose-binding lectin” containing C1qDC gene. **(C)**, A C1qDC gene (LOC111120224) with “galactose-binding lectin” domain was significantly upregulated in *C. virginica* after *P. marinus* challenge.

The globular C1q domain containing (*C1qDC*) proteins are a family of versatile PRRs that has been shown to play a significant role in a variety of processes including immune response, cell adhesion, inflammation, and apoptosis (Wang et al., 2012; Zong et al., 2019). We identified 477 and 333 *C1qDCs* in the genomes of *C. virginica* and *C. gigas*, respectively, and most of these *C1qDCs* belonged to the C1q/Multi-C1q type with two or more C1q domains (Figure 7A). Of note, phylogenetic analysis revealed a small cluster of 24 *C1qDCs* (3 *C. gigas* and 21 *C. virginica*) mostly with the “C1q + galactose-binding lectin” domain that has been shown to play an important role in immune response by binding to endogenous carbohydrates especially from *P. marinus* (Figures 7A,B). Interestingly, these “galactose-binding lectin” containing *C1qDCs* were only found in the genome of *C. virginica* (16 of 21 *CvC1qDC*), not in the *C. gigas* genome (Figure 7A). In addition, a *C. virginica C1qDC* with “galactose-binding lectin” domain (LOC111120224) was significantly upregulated in *C. virginica* after short-term induction of *P. marinus* challenge (Figure 7C). We speculate that this gene plays a significant role in *P. marinus* recognition during early infection stage in *C. virginica*.

### *P. marinus* Responsive *FBGDCs* Are Species Specific

**FIGURE 8:**
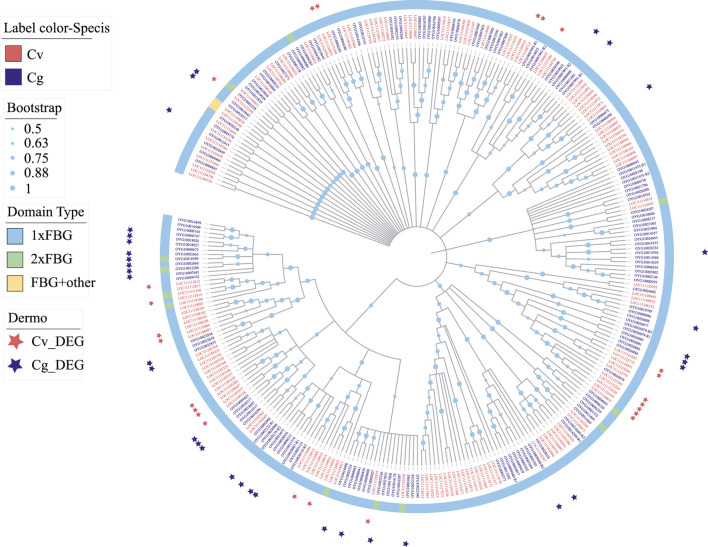
Expansion and phylogeny of FBGDC gene family in *C. virginica* (red font) and *C. gigas* (gray font). The phylogenetic tree is constructed with the maximum likelihood method showing lineage-specific expansion of FBGDC in *C. virginica* (red font) and *C. gigas* (gray font). Different color backgrounds represent different domain composition types, and the red and gray asterisks represent differentially expressed FBGDCs under *P. marinus* challenge in *C. virginica* and *C. gigas*, respectively.

*FBGDCs* (FBG-domain containing proteins) with one or more fibrinogen-like (FBG) domains are important pattern recognition receptors for pathogens recognition in vertebrates (Matsushita et al., 1996), urochordates (Kenjo et al., 2001) and invertebrates (Zhang et al., 2004; Zhang et al., 2009). We detected significant expansion of *FBGDCs* in both *C. virginica* (158 genes) and *C. gigas* (169 genes) (Figure 8). Following *P. marinus* challenge, differential expression was observed in 24 and 30 *FBGDCs* in *C. virginica* and *C. gigas*, respectively (Figure 8). Phylogenetic analysis of the FBG domain showed that most of the *P. marinus* responsive *FBGDCs* were derived from species-specific expansions in the two oyster species (Figure 8). Such results suggested that species-specific expansion of *FBGDCs* may enhance diversity and specificity in PAMP recognitions, and therefore play an important role in the evolution of host-parasite interaction.

## Discussion

The protist *P. marinus* is a lethal pathogen of the eastern oyster *C. virginica*, while its sister species *C. gigas* is strongly resistant. The mechanisms responsible for this remarkable difference in resistance are not understood. In this study, we investigated transcriptomic response to *P. marinus* in two sister species that has diverged for 83 million years (Ren et al., 2010). Our work shows how comparative transcriptomics and genomics analyses contribute to our understanding of evolutionary dynamics of host-parasite interaction lophotrochozoans without adaptative immunity.

Although our discussion below focuses on differences in host defense between the two oyster species, two general observations are worth noting. First, results of this study are highly consistent with a previous study in *C. virginica* (Wang et al., 2010) where DEGs induced by *P. marinus* were determined with a 12 k microarray (challenges were conducted by the same team). For 37.8% of DEGs (389/1,029) from the previous study that had a one-to-one correspondence (BLAST, E-value < 1e-5 and identity >95%) to DEGs of this study (389/759), 83% of theme (323/389) showed the same trend of change in response to *P. marinus* in both studies (Supplemenatary Table S3), indicating that these results are robust. On the other hand, this study and a recent transcriptomic study (Proestou and Sullivan, 2020) shared only 39 DEGs (Supplemenatary Table S4), which could be caused by differences in challenge condition, genetic background or physiological state of oysters used. Second, this study identified 759 and 568 *P. marinus* induced DEGs in *C. virginica* and *C. gigas* (Supplementary Figure S3), respectively, with significant overlapping. The finding of many shared DEGs support a growing body of literature suggesting that there is a conversed set of gene families involved in host defense in molluscs (Gerdol and Venier, 2015; Figueras et al., 2019). In the present study, many immune-related genes (e.g., immune recognition receptors, effector genes, lectins, host-pathogen interaction genes) responded to *P. marinus* exposure in both species. For instance, TLRs are known to play a key role in PAMP recognition, and its activation is the first step in activation of mitogen activated protein kinase (MAPK) and tumor necrosis factor (TNF) during infection of vertebrates, which is pivotal in many cellular processes such as cell growth, differentiation, and apoptosis (Royle et al., 2003). The differential expression of these genes observed in this study is consistent with previous results (Tanguy et al., 2004; Wang et al., 2010), and may reflect a general response or cellular disorder caused by infections.

Our comparative transcriptomics and genomics analyses identified four previously undescribed molecular mechanisms that may account for differences in *P. marinus* resistance in two oyster species and illustrate possible evolutionary dynamics in host-parasite interactions. First, the magnitude of upregulation of the immune-related DEGs (e.g., *TLRs*, *FBGDCs*, *C1qDCs SODs* and *Defensins*) were much higher in *C. virginica* than in *C. gigas.* The stronger response of *C. virginica* is also reflected in the upregulation of serine-type protease inhibitors, which have been shown to inhibit proteases from invading *P. marinus*, limit proliferation of *P. marinus in vitro* and confer resistance (He et al., 2012; Gutiérrez-Rivera et al., 2015). In this study, the GO term for “serine-type endopeptidase inhibitor activity” was enriched in upregulated DEGs of *C. virginica*, not *C. gigas.* All these findings suggest that the host response to *P. marinus* in *C. virginica* is more dramatic. Clearly, *C. virginica* recognizes *P. marinus* as a pathogen and is highly sensitive to its infection. Strong immune response does not equate resistance. Over reactive immune response, whether orchestrated by the host or parasite, can lead to pathology and mortality of the host, as it has been shown in Ostreid herpesvirus infections of the Pacific oyster (He et al., 2012). The susceptibility of *C. virginica* does not mean *P. marinus* is a new pathogen. The fact that *P. marinus* responsive *TLRs* of *C. virginica* has been under positive selection and C1qDCs with the *P. marinus* binding “galactose-binding lectin” domain is found in *C. virginica*, not in *C. gigas* (see below), suggest that the *C. virginica* and *P. marinus* have a long history of coevolution, and *P. marinus* is not a new pathogen of *C. virginica*. If true, the susceptibility and heavy mortality observed in *C. virginica* may be caused by recently increased virulence of *P. marinus* during the coevolutionary race between the host and parasite (Van Valen, 1973). It has been suggested that increased virulence possibly associated with warm winters due to global warming played major roles in outbreaks and northward range extension of *P. marinus* (Ford, 1996; Carnegie et al., 2021).

In contrast to the stronger response of many immune response genes in *C. virginica*, *P. marinus* induced stronger upregulation of *IAP*s in *C. gigas* than in *C. virginica.* Inhibitors of apoptosis play critical roles in immune and stress responses, and the expansion of *IAPs* underpins the remarkable environmental resilience of bivalves (Guo et al., 2015; Song et al., 2021). It has been suggested that, as an intracellular parasite, *P. marinus* induces inhibition of apoptosis in the host as a survival strategy (Hughes et al., 2010). The observation of heightened upregulation of *IAPs* in the resistant *C. gigas* in this study challenges the idea that inhibition of apoptosis of host cells is orchestrated by the parasite. Rather, it suggests that inhibition of apoptosis is initiated by the host as an active defense mechanism and may play a role in limiting parasite proliferation or release, making *C. gigas* less susceptible.

A second mechanism is underscored by dramatic differences in “galactose-binding lectin” domain containing genes between two oyster species. In a previous study (Tasumi and Vasta, 2007), a novel galectin with a unique carbohydrate recognition domain structure had been shown to be associated with the recognition of *P. marinus* in *C. virginica* hemocytes. Galectins is an evolutionarily conserved family of β-galactoside-binding lectins that play important roles in early development and immune response of organisms by binding to endogenous carbohydrates (Rabinovich et al., 2002; Ahmed et al., 2009). In addition, galectins have also been shown to act as PRRs in innate immunity by binding to exogenous glycans on the surface of potentially pathogenic microbes, parasites, and fungi (Kim et al., 2008; Wang et al., 2011; Bao et al., 2013). It is not surprising to find significant upregulation of *galectins* in *C. virginica* but not in *C. gigas.* Further, we annotated 16 “galactose-binding lectin” containing *C1qDCs* in the genome of *C. virginica*, but none in the genome of *C. gigas*. More importantly, a *CvC1qDC* that composed of “galactose-binding lectin” domain was significantly upregulated in *C. virginica* after *P. marinus* challenge. Together, these findings suggest that the presence and upregulation of genes with “galactose-binding lectin” domain in *C. virginica* may be an important factor contributing in its susceptibility to *P. marinus*. Again, these results support the theory that *C. virginica* and *P. marinus* have a long history of coevolution. The lack of galectin upregulation and absence of ‘galactose-binding lectin’ containing *C1qDCs* in *C. gigas* may limit the ability of *P. marinus* to bind and enter host hemocytes. A more complete understanding of the evolution and functional integrations of multigene families in a broad range of host responses and defenses may emerge from further investigations of the innate immune recognition systems found in invertebrate species.

A third notable mechanism rests on the duplication and functional divergence of immune receptors that contributes to differential recognition and response to *P. marinus* in the two oyster species. As more genomes and transcriptomes across diverse marine invertebrates are sequenced, it is becoming clear that gene duplication and subsequent diversification of expanded members of gene families driven by selection are a recognized mechanism of genome innovation and adaptation (Conant and Wolfe, 2008; Zhang et al., 2012; Zhang et al., 2014; Guo et al., 2015; Zhang et al., 2015). In our study, multigene families encoding innate immune receptors, such as *TLRs*, *C1qDCs*, *FBGDCs*, have experienced large-scale expansion and functional diversification in both *C. virginica* and *C. gigas*, which may play an important role in the immune response to different pathogens (Zhang et al., 2015). While the gene families are conserved, distinct gene members of the expanded families are involved in parasite defense in the two oyster species. For example, *TLRs* showed greater expansion in *C. virginica*, and the majority of *TLRs* upregulated by *P. marinus* are derived from distinct species-specific expansions in the two species. Similarly, species-specific expansion was also detected in gene families of *C1qDC* and *FBGDC*, which is consistent with previous findings for major histocompatibility complex (*MHC*) and immune globlin (*Ig*) genes in vertebrates, *TLRs* in sea urchin, *TNFs* in amphioxus and *FREPs* in freshwater snail (Nei et al., 1997; Rast et al., 2006; Huang et al., 2008; Hanington and Zhang, 2010). Moreover, the lineage-specific expansion of immune genes is associated not only with differential responses to pathogens but also with differential expression under environmental stress conditions that emulate those of its natural habitat (Zhang et al., 2015). These findings suggest that lineage-specific expansions of multigene families encoding innate immune receptors in present study may have led to divergence and species-specific functions between *C. gigas* and *C. virginica*.

The fourth mechanism worth noting is that positive selection might be an important driving force for immune recognition of *P. marinus*. Classical evolutionary studies of protein-coding genes have established that genes in the canonical immune system tend to be the most rapidly evolving genes within and among species (Sackton, 2018). Many immune-related molecules directly interact with rapidly evolving pathogenic structures causing strong parasite-mediated natural selection at particular binding positions consistent with the Red Queen hypothesis (Woolhouse et al., 2002). PRRs are evolutionary conserved proteins and believed to be under strongly functional constraint. However, recent studies have found evidence of positive selection in some vertebrate groups, such as positive selection sites (PSSs) in *TLRs* of birds, wild rodents, domestic pig and cetacean (Alcaide and Edwards, 2011; Fornůsková et al., 2013; Darfour-Oduro et al., 2015; Xu et al., 2018). Similarly, positive selection effects on PRRs have also been found in molluscs, including PSSs detected at peptidoglycan recognition proteins (PGRPs) in *C. gigas* (Zhang and Yu, 2013), and fibrinogen-related proteins (FREPs) in the fresh-water snail, *Biomphalaria glabrata* (Hanington and Zhang, 2010). All these studies suggested that positive selection has played an important role in the evolution of metazoan PRRs, which is consistent with co-evolution of host and pathogens (Dodds and Thrall, 2009; Phillips et al., 2018). The *TLRs* of *C. virginica* may have experienced greater selection pressure than that of *C. gigas* based on the positive selection analysis, which suggests that the *TLRs* of *C. virginica* may be actively evolving for recognition of *P. marinus*, while *C. gigas* may possess *TLRs* with more effective defense capability against *P. marinus* infection. This is consistent with the finding that large expansions of *PRRs* are due to lineage-specific selective pressure from pathogens, and pathogen-mediated selection may play a role in the evolution of these genes across a variety of taxa (Hanington and Zhang, 2010; Grueber et al., 2014). As expected, all positive selection sites identified in *P. marinus* responsive *TLRs* of *C. virginica* were located in the extracellular LRR domain that has been shown to be important for pathogen recognition in the host (Khan et al., 2019), consistent with the phenomenon found in Areal’s study (Areal et al., 2011). The evolved *TLRs* may play an important role in the specific recognition of *P. marinus* by *C. virginica*, adding support for coevolution, but further experimental verification is needed.

In summary, comparative analyses in this study revealed previously undescribed molecular mechanisms underlying remarkably differences in *P. marinus* resistance between two sister oyster species. Immune responses to *P. marinus* are more dramatic in *C. virginica*, and the over-reacting immune responses may contribute to increased stress and mortality of the host. In addition, the findings of *C1qDCs* with “galactose-binding lectin” domain that are implicated in *P. marinus* recognition and positive selection in *P. marinus* responsive *TLRs* in *C. virginica* indicate that *C. virginica* and *P. marinus* have a history of coevolution. These observations indicate that *P. marinus* is not a new pathogen of *C. virginica*, and the recent outbreaks may be due to increased virulence of *P. marinus.* Inhibitors of apoptosis showed stronger upregulation in resistant *C. gigas* suggesting it is an active defense of the host, rather than a passive response orchestrated by the parasite as previously suggested. Our results also suggest that gene duplication, functional divergence, and positive selection are important in shaping immune response and host-parasite interaction in the two oyster species. Understanding the molecular basis of host-parasite interaction and coevolution is one of the key goals of immunology, and this study provides an example for using non-model species in comparative analyses.

## Data Availability

All sequencing data associated with this project were deposited in the National Center for Biotechnology Information (NCBI) Sequence Read Archive database, BioProject Accession Numbers: PRJNA778545.

## Supplementary Material

Supplementary Figure 1 Domain structure of galectin **(LOC111130470, Top)** and its upregulation **(Bottom)** under *P. marinus* challenge in *C. virginica*.

Supplementary Figure 2 Phylogenetic tree and positive selection analysis of the TLRs from the largest tandem repeat cluster in *C. gigas.* No positively selected branch or site was detected among these TLRs.

Supplementary Figure 3 Volcano plots of transcriptional changes under short-term and long-term *P. marinus* challenge in *C. gigas.* Blue points represent downregulated genes, red points represent upregulated genes, and black points represent non-differentially expressed genes.
