# Supplementary material for: Transcriptomic Response to Perkinsus marinus in Two Crassostrea Oysters Reveals Evolutionary Dynamics of Host-Parasite Interactions
Source: Front Genet. 2021 Dec 3;12:795706. doi: 10.3389/fgene.2021.795706 (PMC8678459; doi:10.3389/fgene.2021.795706)
Supplement: Supplementary file 3 [file Table1.DOCX]

Tab. 1 Summary of sequencing data for each sample.

|  | Sequenced Reads | Mapped Reads | Alignment Ratio |
| --- | --- | --- | --- |
| *Crassostrea virginica* |  |  |  |
| V0 | 16328214 | 13199156 | 80.80% |
| VC1 | 16598727 | 13984256 | 84.20% |
| VD1 | 16813928 | 13872929 | 82.50% |
| VC2 | 17058041 | 14324702 | 84.00% |
| VD2 | 12307353 | 10156389 | 82.50% |
| *Crassostrea gigas* |  |  |  |
| G0 | 14838062 | 12280738 | 82.80% |
| GC1 | 17054190 | 14304682 | 83.90% |
| GD1 | 15273123 | 12532346 | 82.10% |
| GC2 | 15565037 | 12761073 | 82.00% |
| GD2 | 17203224 | 13954807 | 81.10% |
