# Supplementary figures and images for: Transcriptomic Response to Perkinsus marinus in Two Crassostrea Oysters Reveals Evolutionary Dynamics of Host-Parasite Interactions
Source: Front Genet. 2021 Dec 3;12:795706. doi: 10.3389/fgene.2021.795706 (PMC8678459; doi:10.3389/fgene.2021.795706)

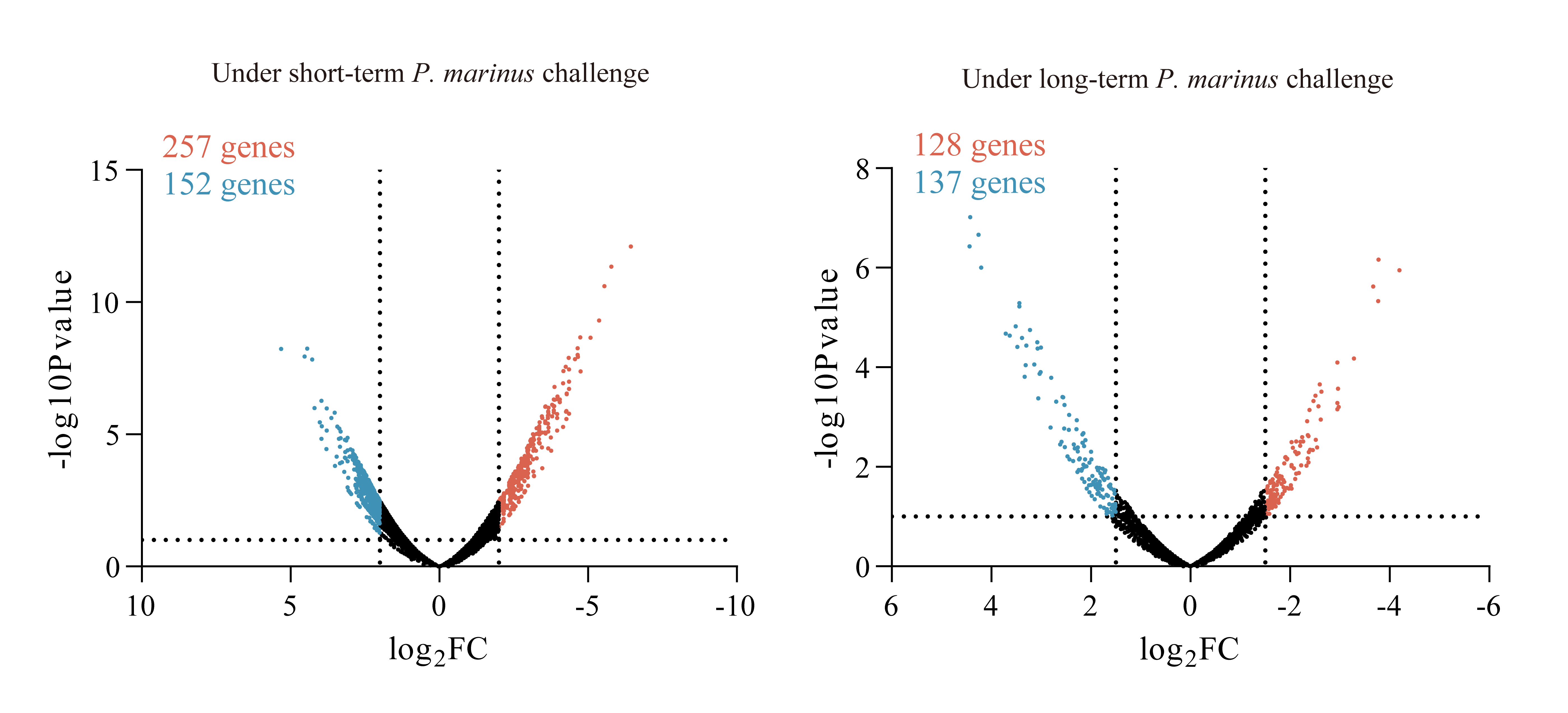

Supplement: Supplementary file 4 [file Image3.TIF]

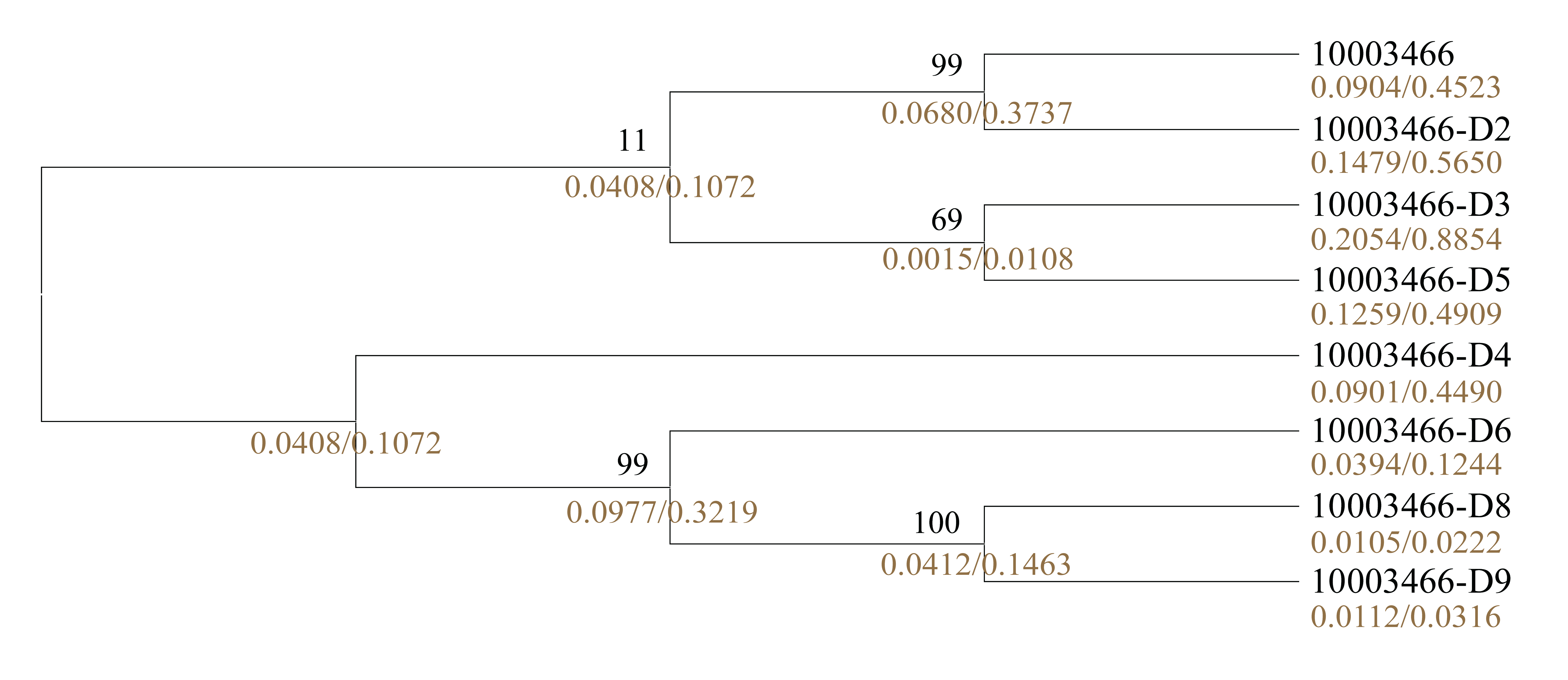

Supplement: Supplementary file 5 [file Image2.TIF]

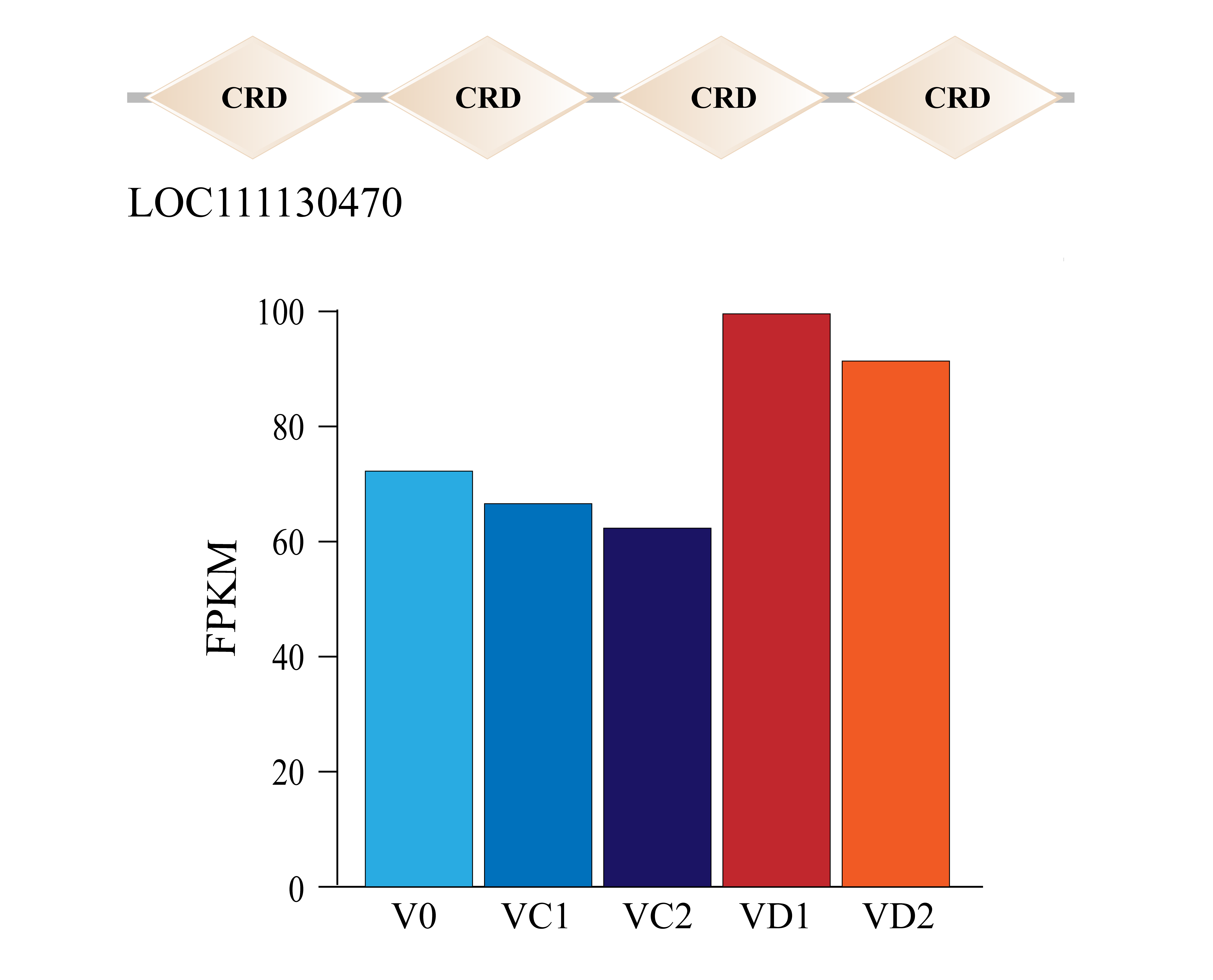

Supplement: Supplementary file 6 [file Image1.TIF]
